# Time-Dependent Changes in Death Reports and the Sex Ratio in the Safety Surveillance of SARS-CoV-2 Vaccination in Japan, the United States, and European Countries

**DOI:** 10.7759/cureus.23380

**Published:** 2022-03-22

**Authors:** Erika Yamashita, Morihito Takita, Masahiro Kami

**Affiliations:** 1 Research Division, Medical Governance Research Institute, Minato-ku, Tokyo, JPN

**Keywords:** older adult, adverse event, open database, safety surveillance, covid-19 vaccines

## Abstract

The national safety surveillance of vaccines is a fundamental measure to ensure vaccination safety and maintain transparency and public trust. Our previous study revealed an early increase in death reports after the severe acute respiratory syndrome coronavirus 2 (SARS-CoV-2) vaccination in Japanese surveillance despite our hypothesis that no time-dependent variations in the number of death reports would be seen if the vaccination is not related to serious adverse events. This study is an extensive investigation to determine whether the number of death reports varied consistently over time after vaccination in the older population in Japan, the US, and European countries. We collected the death reports after BNT162b2 mRNA vaccination in individuals aged 65 years or older using the open databases in Japan, the US (Vaccine Adverse Event Reporting System, VAERS), and European countries (EudraVigilance). We observed an early increase of death reports on Day 2 after the vaccination in all three databases. The female-to-male ratio was also assessed and showed a certain degree of time-dependence (*R^2^* of linear regression 0.54, *p* =0.01) in Japan but not in the US and European countries. The findings suggest the existence of unknown predictors of the adverse events of the SARS-CoV-2 vaccination, especially for the older Japanese population. The continuous and careful monitoring safety aspects of the vaccines are warranted.

## Introduction

The administration of severe acute respiratory syndrome coronavirus 2 (SARS-CoV-2) vaccine booster doses has expanded in Europe, North America, and the Middle East. In Israel, they have initiated the second booster (fourth dose) administration for older people in response to further waves of SARS-CoV-2 infection [1-2]. In Japan, the vaccine coverage of the primary series achieved 77.8% at the end of December 2021, and booster administration began for older people and medical professionals in December 2021. The completion of primary vaccination in developing countries has been strongly urged [3-4].

The risk-benefit ratio of SARS-CoV-2 vaccination has been considered favorable to expand the vaccination broadly [5-7]. A concern on significant but rare adverse events such as myocarditis after mRNA vaccination in adolescents was raised after approval of emergency use by regulatory authorities [8-9]. The initial clinical trials for the approval process commonly include healthy individuals or those with stable disease conditions [10], which may miss adverse events in patients at risk. Thus, post-authorization safety surveillance should be carefully maintained to find unknown risks of SARS-CoV-2 vaccination like the other existing vaccines. The national safety surveillance of vaccines such as the Vaccine Adverse Event Reporting System (VAERS) in the United States (US) and EudraVigilance in European countries is a fundamental measure to ensure vaccination safety and maintain transparency and public trust towards mass vaccination [11-12]. In Japan, the Preventive Vaccination Act regulates the national safety surveillance of vaccines [13].

Our past study revealed a peak of death reports on Day 2 after the vaccination despite our hypothesis that there would be no time-dependent variation in the number of deaths after the SARS-CoV-2 vaccination if the vaccination is not related to serious adverse events [14]. The finding suggests a possibility to overlook individuals vulnerable to adverse events of the vaccination and warrants the continuous efforts of the vaccine safety surveillance. This study expanded safety data in Japan up to October 2021 and added the Vaccine Adverse Event Reporting System (VAERS) data in the US and EudraVigilance in European countries. This study focused on the older population since they are susceptible to SARS-CoV-2 infection [15-16]. We kept our hypothesis that there would be no time course-dependent variations after SARS-CoV-2 vaccination in the number of deaths and sex ratio if the vaccination is not associated with serious adverse events.

## Materials and methods

Data collection on death reports in vaccine safety surveillance in Japan, the US, and European countries

We collected death reports after coronavirus disease (COVID) vaccination that were publicly available by the Ministry of Health, Labour and Welfare of Japan, VAERS in the US, and EudraVigilance in European countries [17-19]. This study focused on the death reports of individuals aged 65 years or older and those vaccinated with the BNT162b2 mRNA vaccine (Research Division, Medical Governance Research Institute, Minato-ku, Tokyo, JPN). The cases reported between February 17, 2021, and October 15, 2021, in Japan, December 21, 2020, and December 2, 2021, in VAERS, and December 24, 2020, and December 10, 2021, EudraVigilance were included in this study. The cases were excluded if the vaccine administered, date of vaccination, vaccination doses, age, or sex were not available in Japan and the US. The vaccine doses were not available in the EudraVigilance; thus, we evaluated cases with the vaccine administered, date of vaccination, age, and sex for European countries. The duplicate cases were excluded after determining identifiers in the databases.

Ethical considerations

The study was exempt from ethical review in terms of the Ethical Guidelines for Medical and Biological Research Involving Human Subjects in Japan since we used publicly available data.

Analysis

We performed descriptive analysis on the number of death reports over time along with the stratification of sex and vaccine administration doses. We calculated the time between the dates of vaccination and death and defined the date of vaccination as Day 1. The relationship between the female-to-male ratio and days after vaccination was visualized to evaluate whether the sex ratio depended on the time course. Linear regression models were employed to investigate the time-course dependence of the sex ratio. Chi-square tests were performed to compare the sex proportions of the two groups classified by the post-vaccination duration. The monthly sex ratio of deaths in the general population aged 65 years or older in Japan was visualized using the 2019 census data. This assessment was done to see the time variations since the mass vaccination was conducted in July and August 2021 for the older population in Japan [20]. The statistical evaluation was performed with GraphPad Prism 9 (GraphPad Software Inc., San Diego, CA). The statistical significance was defined as two-sided *p* values <0.05.

## Results

Japan

**Figure 1:**
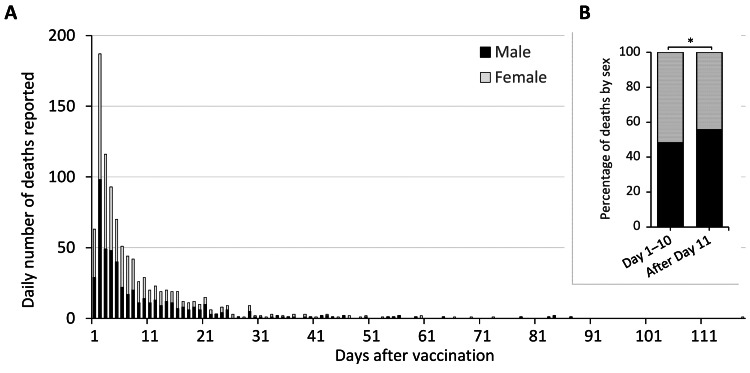
The daily number of death reports after BNT162b2 mRNA vaccination in Japan The daily number of death reports after BNT162b2 mRNA vaccination in the Japanese vaccine surveillance system is shown. The number was classified by sex: male (dark) and female (gray) (A). We compared the sex proportion before and after Day 10 post-vaccination (B), which showed a statistically significant difference (*p =0.037).

**Figure 2:**
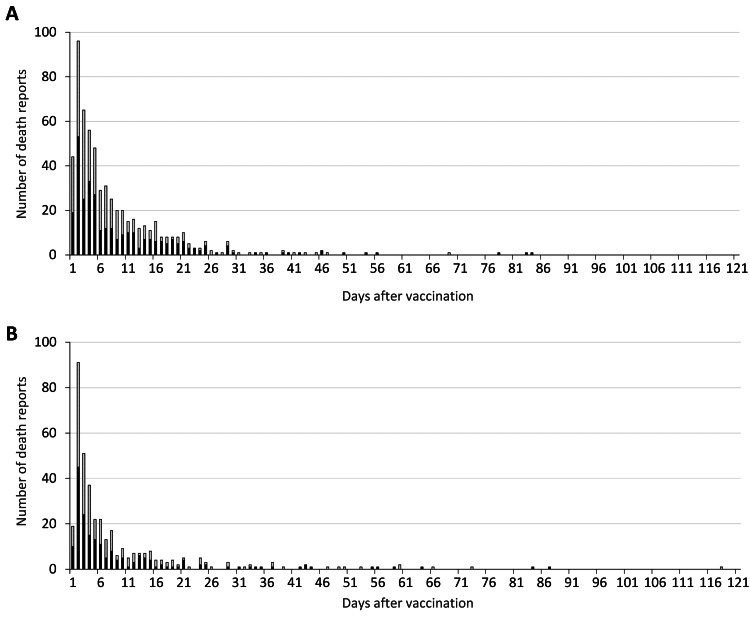
Daily number of death reports after vaccination classified by dose in Japan The daily number of death reports after BNT162b2 mRNA vaccination was shown after classifying by the first (A) and second (B) doses. The number was classified by sex: male (dark) and female (gray).

We identified a total of 994 death reports, consisting of 500 males and 494 females, after BNT162b2 mRNA vaccination during the study period (Table 1). The highest daily number of death reports (*n* = 187) was found on Day 2 (Figure 1). The number immediately declined to nine cases or fewer after Day 22. The same pattern was observed after classifying the first and second doses (Figure 2). Of note, the BNT162b2 mRNA vaccine was given to 31,998,666 and 31,674,719 older people, excluding medical professionals, for first and second doses by October 15, 2021 [21]. We could not find statistics on sex stratification among the BNT162b2 mRNA vaccine recipients.

**Table 1:** Characteristics of death reports after BNT162b2 mRNA vaccination in national safety surveillance Characteristics of death reports after BNT162b2 mRNA vaccination in national safety surveillance are shown Abbreviation: N.A.: data not available, US: United States.

Country	Japan	US	European countries
Number of death reports	994	2,305	1,384
Age (years) –median [interquartile range]	84 [77–89]	81 [73–87]	N.A.
Sex-Female –number (percentage)	494 (50)	1,030 (45)	738 (53)
Number of death reports after classifying dose of vaccine administration –number (percentage)			
First dose	609 (61)	761 (33)	N.A.
Second dose or more	385 (39)	1,543 (67)	N.A.
Days post-vaccination –median [interquartile range]	5 [2–12]	36 [9–152]	3 [2–9]

**Figure 3:**
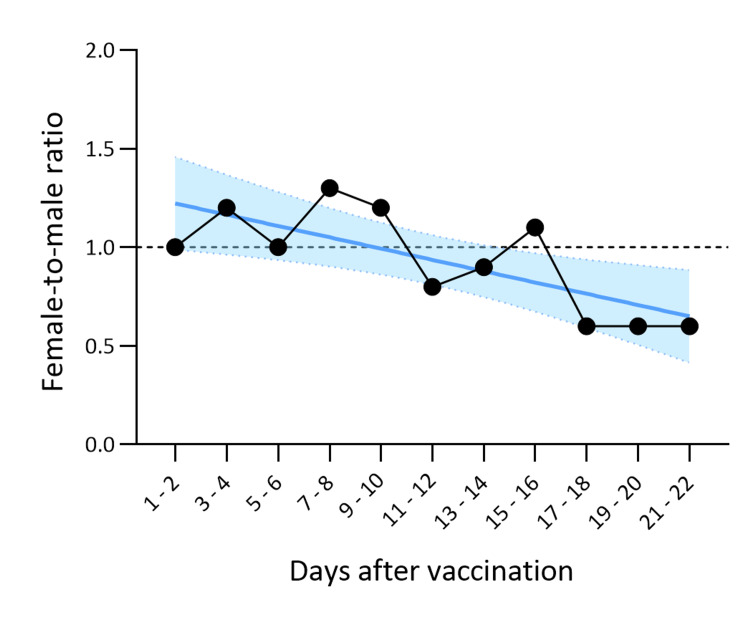
Changes in the sex proportion in death reports after vaccination in Japan The female-to-male ratio in the death reports of the safety surveillance system in Japan was plotted with days after vaccination (line with circles). Variations in the female-to-male ratio can fit with a linear regression (blue line) (*R^2^* =0.54, *p* =0.01). The blue shadow area indicates a 95% confidence interval.

Then, we assessed changes in the sex ratio of females to males in the number of death reports every two days between Day 1 and Day 22 post-vaccination (Figure 3). The changes followed a simple linear regression (R2 =0.54, *p* =0.01), which indicates that variations in the sex ratio can be a time-dependent variable. We also confirmed the statistically significant difference in the sex proportion of death reports before and after Day 10 of vaccination; a significantly higher percentage of females were observed from Days 1 to 10 when compared with the post-Day 11 period (51.7% and 44.3% in females, respectively, *p* =0.037) (Figure 1).

**Figure 4:**
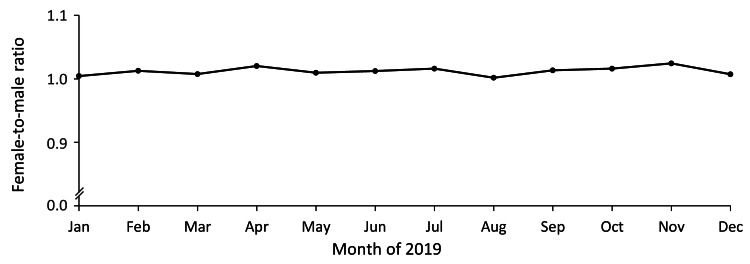
No time-dependent changes in the sex ratio of deaths in people aged 65 years or older in Japan The monthly female-to-male ratio in the population aged 65 years or older in Japan is shown. The sex ratio remained 1.0 across the year.

We investigated the sex ratio in the number of deaths in the general population aged 65 years or older in Japan since time-dependent changes might influence the sex ratio of death reports due to mass SARS-CoV-2 vaccination from July to August 2021. However, the sex ratio remained 1.0 across the year (Figure 4).

The US and Europe

**Figure 5:**
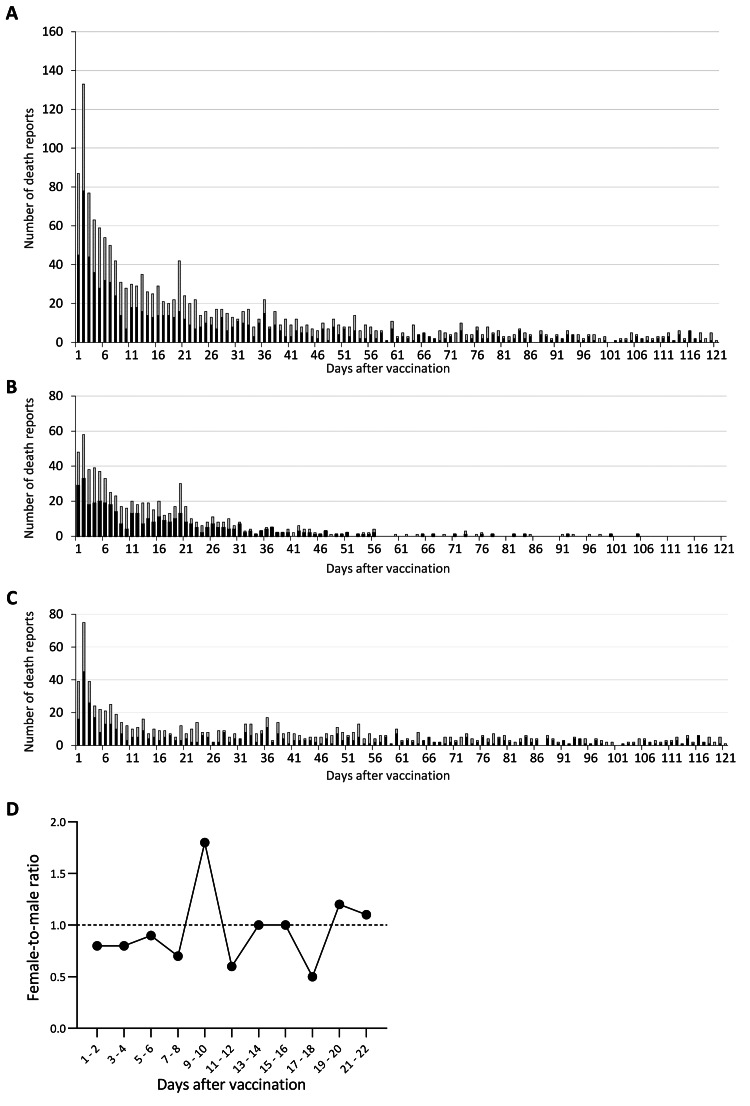
Number of death reports after BNT162b2 mRNA vaccination in the VAERS The number of death reports after BNT162b2 mRNA vaccination in the Vaccine Adverse Event Reporting System (VAERS) is shown as a total (A), after classifying the first (B) and after the second or more doses (C). The bars were painted dark and gray for males and females, respectively. The female-to-male ratio of deaths after vaccination reported in VAERS is also shown (D). No trend of linear regression was found for the variations in the sex ratio.

We identified a total of 2,305 death reports, consisting of 1,275 males (55%) and 1,030 females (45%), after BNT162b2 mRNA vaccination in VAERS (Table 1). The number of death reports peaked on Day 2 post-vaccination (Figure 5A). The same pattern was observed even after stratifying for the first and second or more doses (Figures 5B-5C). We investigated the time-dependence of the sex ratio of death reports after the vaccination and did not observe any time-course changes (Figure 5D).

**Figure 6:**
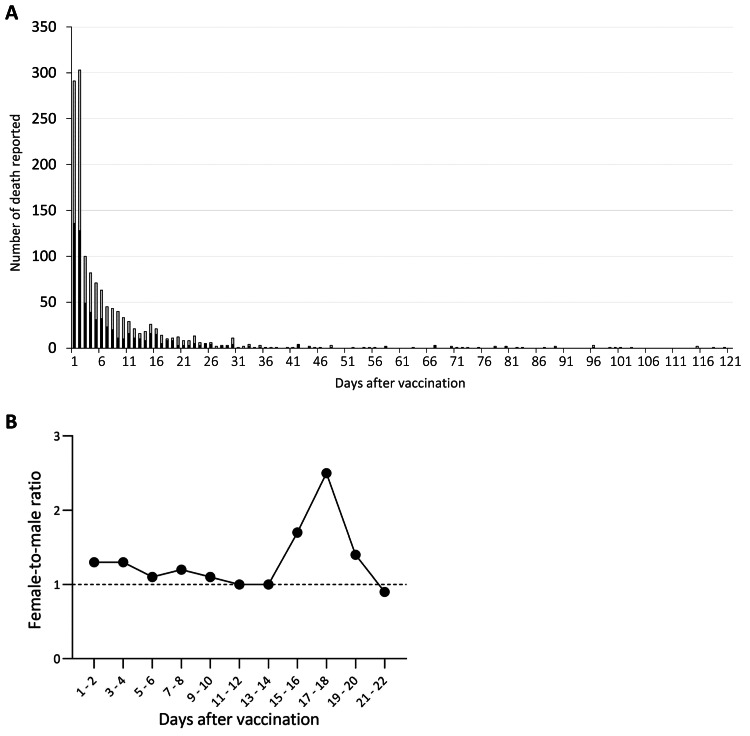
Number of death reports after BNT162b2 mRNA vaccination in EudraVigilance The number of death reports (A) and the female-to-male ratio (B) after BNT162b2 mRNA vaccination in EudraVigilance are shown. The number was classified by sex: male (dark) and female (gray). No linear regression trend was found for the variations in the sex ratio.

We referred to the European data in EudraVigilance and found a total of 1,384 death reports after BNT162b2 mRNA vaccination, consisting of 646 males (47%) and 738 females (53%) (Table 1). The peak of death reports was seen on Day 2, and the number declined to one-tenth after Day 11 of vaccination (Figure 6A). The doses of vaccination were not available in EudraVigilance. No association of linear regression was detected between the female-to-male ratio and the time after vaccination (Figure 6B).

## Discussion

In this study, we observed the increase of death reported in the first few days after BNT162b2 mRNA vaccination in the older population in the national safety surveillance of Japan, the US, and European countries, consistently with our previous study [14]. We also revealed time-dependent changes in sex ratio in the death reports in Japan, although no such variations were found in the US and European databases. The findings, however, are carefully interpreted since there are several limitations due to the characteristics of the national safety surveillance. We may still miss the unknown risk of the current regimen of SARS-CoV-2 vaccination for older people in Japan and should keep careful monitoring of adverse events of SARS-CoV-2 vaccines.

There are some possible reasons behind the variations in the sex ratio in the death reports after the vaccination in the older Japanese population. Adverse events might develop weight- and dose-dependently [22-23]. We observed a significantly higher proportion of death reports of females from Days 1 to 10 when compared to the period after Day 11. The body mass index in the Japanese female population is smaller than in the US and European countries [24]. Their smaller weight might influence the post-vaccination conditions. Thus, dose adjustment may need to be considered if the body weights of vaccine recipients are significantly lighter. In fact, the children aged 11 years or younger are received a reduced amount [25]. Recipient characteristics, such as body weight, are not available in the open database of safety surveillance, making it difficult to determine the causal relationship. Another reason is a sex difference in acute adverse events in which females might be associated [26-27]. Lastly, a reporting bias exists as described later.

In the original clinical trial of the BNT162b2 mRNA vaccine, 15 and 14 deaths were observed in the intervention (*n* =21,926) and placebo (*n* =21,921) groups, respectively [5]. The investigators assessed that there was no death related to BNT162b2 mRNA vaccination. Safety monitoring is continued up to two years after the second dose of the vaccine. An early study assessing SARS-CoV-2 vaccine safety with VAERS pointed out that older people and males were more likely to report death and serious adverse events [28]. The final evaluation of safety data of the BNT162b2 mRNA vaccine has not been completed yet.

This study has several limitations. The first is the lack of a placebo control group. Second, we considered the reporting bias [11]. The Japanese vaccine safety surveillance allows only physicians to report the adverse events, whereas VAERS and EudraVigilance accept the submission by the recipients and their families. Underestimation will happen when busy physicians miss reporting the adverse events. Third, there is difficulty in predicting the incidence of adverse events, including death after vaccination, because the safety database is separated from the vaccine recipient database. Therefore, we could not assess the death rates directly; instead, we evaluated if the death reports would show the uneven distribution after vaccination to assume the existence of SARS-CoV-2 vaccine risks. We estimated that a death report was submitted approximately every 5.3 × 10^4^ and 8.2 × 10^4^ administrations of the first and second doses in Japan by calculation with the two separated data sources. Though the frequency should be carefully interpreted, there is a gap in the data collection between the safety and vaccine administration databases, and not all death reports were related to the vaccinations. In New York states, they connected multiple databases of vaccine administration, electronic health records, and laboratory data and applied them to estimate the vaccine efficacy [29]. This mechanism may help assess the safety of vaccination and find recipient characteristics to associate with adverse events. Fourth, some countries lack access to the individual level of death reports; for example, the summarized data are publicly available in the UK but not for individual information [30].

## Conclusions

In conclusion, we found an early peak of death reports in older individuals in the days following BNT162b2 mRNA vaccination in Japan, the US, and Europe. We also observed time-dependent changes in the sex ratio of the deceased after the vaccination reported in Japan. These findings suggest that we may be unaware of specific characteristics that increase susceptibility to the serious adverse events of SARS-CoV-2 vaccination. The careful and continuous monitoring of vaccine safety is warranted.

## References

[1] Shekhar R, Garg I, Pal S, Kottewar S, Sheikh AB (2021). COVID-19 vaccine booster: to boost or not to boost. Infect Dis Rep.

[2] Richterman A, Scott J, Cevik M (2021). Covid-19 vaccines, immunity, and boosters. BMJ.

[3] Hassan F, London L, Gonsalves G (2021). Unequal global vaccine coverage is at the heart of the current covid-19 crisis. BMJ.

[4] The Lancet Infectious Diseases (2021). COVID-19 vaccine equity and booster doses. Lancet Infect Dis.

[5] Thomas SJ, Moreira ED Jr, Kitchin N (2021). Safety and efficacy of the BNT162b2 mRNA Covid-19 vaccine through 6 months. N Engl J Med.

[6] El Sahly HM, Baden LR, Essink B (2021). Efficacy of the mRNA-1273 SARS-CoV-2 vaccine at completion of blinded phase. N Engl J Med.

[7] Bansal A (2022). Vaccine equity: there is no time to waste. Bull World Health Organ.

[8] Mevorach D, Anis E, Cedar N (2021). Myocarditis after BNT162b2 mRNA vaccine against Covid-19 in Israel. N Engl J Med.

[9] Baden LR, El Sahly HM, Essink B (2021). Efficacy and safety of the mRNA-1273 SARS-CoV-2 vaccine. N Engl J Med.

[10] Polack FP, Thomas SJ, Kitchin N (2020). Safety and efficacy of the BNT162b2 mRNA Covid-19 vaccine. N Engl J Med.

[11] Shimabukuro TT, Nguyen M, Martin D, DeStefano F (2015). Safety monitoring in the Vaccine Adverse Event Reporting System (VAERS). Vaccine.

[12] Postigo R, Brosch S, Slattery J (2018). EudraVigilance medicines safety database: publicly accessible data for research and public health protection. Drug Saf.

[13] Noda A, Sakai T, Tsuchiya M, Oyanagi G, Obara T, Mano N (2020). Characteristics of adverse events following immunization reporting in children: the Japanese Adverse Drug Event Report Database. Vaccines (Basel).

[14] Yamashita E, Ozaki A, Tsubokura M, Takita M, Tanimoto T, Kami M, Rodriguez-Morales AJ (2021). Safety of SARS-CoV-2 BNT162b2 vaccine in elderly patients from Japan - a preliminary assessment and a call on careful pharmacovigilance. Revista Panamericana de Enfermedades Infecciosas.

[15] Williamson EJ, Walker AJ, Bhaskaran K (2020). Factors associated with COVID-19-related death using OpenSAFELY. Nature.

[16] CDC COVID-19 Response Team (2020). Severe outcomes among patients with coronavirus disease 2019 (COVID-19) - United States, February 12-March 16, 2020. MMWR Morb Mortal Wkly Rep.

[17] (2021). Summary of cases reported as deaths after vaccination with new corona virus. https://www.mhlw.go.jp/content/10601000/000846547.pdf.

[18] (2022). VAERS data sets. https://vaers.hhs.gov/data/datasets.html.

[19] Agency Agency, E.M E.M (2022). EudraVigilance: electronic reporting. https://www.adrreports.eu/index.html.

[20] (2019). Demographic statistics in 2019 census. https://www.e-stat.go.jp/dbview?sid=0003411687.

[21] (2022). The total number of COVID-19 vaccine administrations. https://www.kantei.go.jp/jp/content/vaccination_data5.pdf.

[22] Walsh EE, Frenck RW Jr, Falsey AR (2020). Safety and immunogenicity of two RNA-based Covid-19 vaccine candidates. N Engl J Med.

[23] Anderson EJ, Rouphael NG, Widge AT (2020). Safety and immunogenicity of SARS-CoV-2 mRNA-1273 vaccine in older adults. N Engl J Med.

[24] NCD Risk Factor Collaboration (NCD-RisC) (2016). Trends in adult body-mass index in 200 countries from 1975 to 2014: a pooled analysis of 1698 population-based measurement studies with 19·2 million participants. Lancet.

[25] Walter EB, Talaat KR, Sabharwal C (2022). Evaluation of the BNT162b2 Covid-19 vaccine in children 5 to 11 years of age. N Engl J Med.

[26] CDC COVID-19 Response Team, Food and Drug Administration (2021). Allergic Reactions Including Anaphylaxis After Receipt of the First Dose of Pfizer-BioNTech COVID-19 Vaccine - United States, December 14-23, 2020. MMWR Morb Mortal Wkly Rep.

[27] Almohaya AM, Alsubie H, Alqarni B, Alzayad B, Alghar A, Alshahrani K, Barry M (2022). Acute unsolicited adverse events following BNT162b2 vaccine in Saudi Arabia, a real-world data. Vaccine.

[28] Xiong X, Yuan J, Li M, Jiang B, Lu ZK (2021). Age and gender disparities in adverse events following COVID-19 vaccination: real-world evidence based on big data for risk management. Front Med (Lausanne).

[29] Rosenberg ES, Dorabawila V, Easton D (2022). Covid-19 vaccine effectiveness in New York State. N Engl J Med.

[30] (2021). Report of the Commission on Human Medicines Expert Working Group on COVID-19 vaccine safety surveillance. https://www.gov.uk/government/publications/report-of-the-commission-on-human-medicines-expert-working-group-on-covid-19-vaccine-safety-surveillance.

